# Intensive Care Unit Admission after Cytoreductive Surgery and Hyperthermic Intraperitoneal Chemotherapy. Is It Necessary?

**DOI:** 10.1155/2014/307317

**Published:** 2014-04-22

**Authors:** Horacio N. López-Basave, Flavia Morales-Vasquez, Carmen Mendez-Herrera, Silvio A. Ñamendys-Silva, Kuauhyama Luna-Ortiz, German Calderillo-Ruiz, Jesús Cabrera Rojas, Erika Ruiz-Garcia, Angel Herrera-Gomez, Juan M. Ruiz-Molina, Abelardo Meneses Garcia

**Affiliations:** ^1^Department of Gastroenterology, National Cancer Institute, San Fernando No. 22, Colonia Seccion XVI, 14050 México City, DF, Mexico; ^2^Department of Medical Oncology, National Cancer Institute, San Fernando No. 22, Colonia Seccion XVI, 14080 México City, DF, Mexico; ^3^Department of Embryology at the National Autonomous University of Mexico (UNAM), Insurgentes Sur s/n, Ciudad Universitaria, Colonia Copilco Universidad, 04360 México City, DF, Mexico; ^4^Department of Critical Care Medicine, National Cancer Institute, San Fernando No. 22, Colonia Seccion XVI, 14080 México City, DF, Mexico

## Abstract

*Introduction.* Cytoreductive surgery (CS) with hyperthermic intraperitoneal chemotherapy (HIPEC) is a new approach for peritoneal carcinomatosis. However, high rates of complications are associated with CS and HIPEC due to treatment complexity; that is why some patients need stabilization and surveillance for complications in the intensive care unit. *Objective.* This study analyzed that ICU stay is necessary after HIPEC. *Methods.* 39 patients with peritoneal carcinomatosis were treated according to strict selection criteria with CS and HIPEC, with closed technique, and the chemotherapy administered were cisplatin 25 mg/m^2^/L and mitomycin C 3.3 mg/m^2^/L for 90-minutes at 40.5°C. *Results.* 26 (67%) of the 39 patients were transferred to the ICU. Major postoperative complications were seen in 14/26 patients (53%). The mean time on surgical procedures was 7.06 hours (range 5−9 hours). The mean blood loss was 939 ml (range 100–3700 ml). The mean time stay in the ICU was 2.7 days. *Conclusion.* CS with HIPEC for the treatment of PC results in low mortality and high morbidity. Therefore, ICU stay directly following HIPEC should not be standardized, but should preferably be based on the extent or resections performed and individual patient characteristics and risk factors. Late complications were comparable to those reported after large abdominal surgery without HIPEC.

## 1. Introduction

Cytoreductive surgery (CRS) with intraperitoneal (i.p.) chemotherapy and hyperthermia (HIPEC) has emerged as a novel approach for peritoneal carcinomatosis. This is a complex procedure that implies extensive resection of the peritoneal surface, sometimes multiple visceral resections, high rates of i.p. chemotherapy with hyperthermia, and prolonged operative time (in general, from 10–14 hours). High rates of potential fatal complications associated with HIPEC have been reported in the literature [1, 2]; that is why some patients need to be admitted to intensive care unit for stabilization, detection, and early resolution of complications associated with the extension of the surgical procedure, the toxicity of the drugs administered, or both. Particular emphasis should be placed on the dose of cisplatin administered i.p., because in a multivariate analysis it has been reported that doses >240 mg correlate with the appearance of postoperative complications [3–5]. Post-HIPEC morbidity rates range from 30 to 74% and mortality ranges from 0 to 19% [1, 2, 6, 7]. It is difficult to compare clinical results with others centers in terms of patient selection, surgical technique, time, duration, and degree of hyperthermia, as well as the dosages of the drugs. All factors have been compiled in complication classification systems of morbidity, toxicity, and mortality such as the Clavien-Dindo classification, the Elias classification, the National Cancer Institute (NCI), and the Common Terminology Criteria for Adverse Events (CTCAE). These systems are different and there is correlation among the degrees of complication; thus, the seriousness of a clinical condition does not refer to the clinical severity in another classification [8, 9]. Despite the limitations, taking into account the serious complications that merit a reintervention, admittance to the intensive care unit (ICU), or the utilization of invasive procedures, complication rates range between 12 and 54% [1, 2, 6, 7, 10]. However, it is not clear whether a stay in the ICU is necessary for strict surveillance after CS + HIPEC.

The objective of this work was to analyze whether postoperative management after CS + HIPEC requires postsurgical care in the ICU as a mandatory measure in our Institution.

## 2. Materials and Methods 

We review retrospectively the charts of 39 patients with peritoneal carcinomatosis who were operated on from January, 2007, to January, 2012, after cytoreductive surgery (CRS) with HIPEC with 25 mg/m^2^/L of Cisplatin and 3.3 mg/m^2^/L of Mitomycin C (MMC) administered for 90 min at 40.5°C. The following data were procured: histology; age; gender, date and days of admittance to the ICU, the presence of bleeding, complications, time, and management of complications.

## 3. Results

Of the 39 patients treated with CS and HIPEC technique, 30 were females and 9 males, 14 patients with colorectal cancer, 6 with peritoneal pseudomixoma, 14 with carcinomatosis of the ovary, 2 with gastric cancer, and 3 with cancer of the appendix. The mean age of the patients was 55.4 years (range 30–72 years). The mean time of the surgical procedure was 7 (range 5–10 hours), the mean blood loss was 938.88 mL (range 100–3,700 mL) (Table 1), and 26 (67%) of cases were admitted to the ICU and the mean time in the ICU was 2.7 days (range 1–13 days).

The most frequent complication was diaphragmatic opening (see Table 2). The criteria to admission ICU were prolonged time during surgery and/or blood loss during surgery. There was no difference in complications or mortality between patients in the ICU or out of ICU (Tables 3 and 4). 23 (58%) patients were alive without evidence of disease, seven (18%) were alive with tumor activity, six (15%) died with tumor activity, and three (7.5%) are dead without tumor activity.

## 4. Discussion

Peritoneal carcinomatosis is considered the most common cause of death of intra-abdominal origin [6]. Despite the improvement of the treatment for this disease, CS with HIPEC need of an specialized team, adequate technology and infrastructure, and technological facilities to reduce morbidity and improve quality of life [7]. Likewise, identification of risk factors that increase morbility is also crucial for improving the results. In our study, morbility was 48.6%, the most common complication was diaphragmatic opening (15%), and mortality was 5% (Table 2). It has been described that morbility and mortality are directly proportional to the degree of cytoreduction, the learning curve, and the surgical technique [11, 12].

The complications were similar in severity in UCI and out UCI and during surgery (Table 3). The first two patients did not require admittance into the ICU, and the dehiscence developed 4 days after the patient's admittance into the ICU. In addition one patient developed pneumonia and 3 acute kidney failures, both resolved with medical management (Table 4). Two cases (5%) die due to a postoperative bleeding, identified in the first 4 hours of the patient admittance to the ICU, and the other due to pulmonary thromboembolism, which presented at 48 h of the patient's admittance into the ICU. The rates reported for morbility and mortality range between 0% and 40% and 0 and 12.5%, respectively [13–19] (Table 5).

Smeenk et al. in 2006 [20] reported a toxicity of 54% and a mortality of 3% in 103 peritoneal pseudomixoma procedures, demonstrating the significant association between age and toxicity and intestinal perforation and tumor volume (Table 6).

The present work reports higher mortality when compared with previous studies [1, 2, 19, 20], which can be related to the fact that patients presented a more voluminous tumor disease at the time of surgery; thus, surgical time was longer than in those in whom there was more blood loss and frequency of diaphragmatic opening, which was the site where the greatest tumor burden was localized [19, 20].

Among the causes of death found in the literature were intestinal perforation, dehiscence of the anastomosis, intestinal fistula, bile duct leakage, postoperative bleeding, pancreatitis, and the habitual risks of surgery, such as deep vein thrombosis, pulmonary embolism, pneumothorax, myocardial infarct, bone marrow aplasia, and hematological toxicity. The complications can be associated with the surgery, the hyperthermia, and the chemotherapy. The complications presented in our study do not diminish with the stay in intensive care, and the mortality was similar [21–24].

The gastrointestinal and respiratory tracts were most affected. After the gastrointestinal tract, the respiratory tract is probably the system that is most affected by postoperative complications. Pulmonary morbidity was found in six cases of our series and the majority of these were resolved without reintervention or invasive procedures, with the exception of a case of pulmonary embolism [25].

A study at Wake Forest University reports thoracic complications in a series of 42 patients treated with CS + HIPEC [26]. Thoracic complications were observed in 36 (86%) patients, atelectasia in 32 patients, and pleural effusion in 27 (64%) patients. The majority of the effusions (74%) occurred 1–3 days after CS + HIPEC. The incidence of thoracic complications in the HIPEC group was significantly higher than in the control group (*P* < 0.05). In our study, we uncovered common findings, including bibasilar atelectasia and pleural effusion after the use of MMC, but the majority did not merit any intervention. The prevention and management of these complications included careful inspection of the integrity of the diaphragmatic muscle and resection of its peritoneum. Early repair of eventual macroscopic perforations and prophylactic insertion of thoracic catheters after cytoreduction are practices performed by some authors. With regard to nephrotoxicity, our study reported two cases of alteration of serum creatinine, which after a mean period of 16 days (range 7–42 days) after surgery showed normal kidney function.

Studies reporting the systemic toxicity of CS + HIPEC are resumed up in Table 6. Verwaal reported kidney failure in 4.9% of cases. Glehen et al. observed a postoperative kidney failure rate of 1.3% [27].

The frequent complications found in the majority of series are digestive fistulae, whether in the form of anastomotic leakage or intestinal perforation outside of the anastomosis. Fistulae have been reported in between 3.9 and 34% of patients [17, 18, 28, 29] (Table 5). These numbers are higher than the rate reported for common elective surgery [30, 31].

## 5. Conclusions 

Cytoreduction with intraperitoneal chemotherapy with hyperthermia is a treatment with high morbility. Therefore, adequate selection of patients is very important to diminish the complications that can be associated with the surgery, the hyperthermia, the chemotherapy, or altogether. The results of the present work suggest that the main factor associated with the development of complications is the extension of the CR process and not the application of chemotherapy and hyperthermia as principal factors, given that the delayed complications reported in our study were comparable with those reported in the literature after major abdominal surgery without HIPEC.

The results and mortality of the patients who went on to the ICU and those without the ICU are similar. Admittance to the ICU should be evaluated case by case considering the individual characteristics of the patients, their risk factors, and the extension of their surgical procedure.

## Figures and Tables

**Table 1:** Patient's characteristic.

Variables	No (%)
Age (years)	
Mean	44.5 (30–72)
Gender	
Female	30 (77)
Male	9 (23)
Operative Time	7 hours (5–10 hrs)
Primary site	
Colorectal	14 (35.8)
Ovarian epithelial cancer	14 (35.8)
Pseudomyxoma	6 (15.3)
Appendix	3 (7.6)
Gastric	2 (5)
Previous surgery	39
Previous systemic chemotherapy	30 (77)
PCI	12.8 (2–33)
<20	22 (57%)
>20	17 (43%)

PCI: peritoneal cancer index.

**Table 2:** Complications.

	*N*	%
Diaphragm opening	6	15.36
Fistula	3	7.68
Acute renal failure	3	7.68
Packaging	2	5.12
Pneumonia	1	2.56
Bleeding postoperative	1	2.56
Anastomotic Leak	1	2.56
Mortality	**2**	5.12

Total	*N* = 19	48.6%

Reoperation	**4 cases** (10%)	3 Bleeding into operated site 1 Anastomotic dehiscence

**Table 3:** Complications and mortality by place.

Site	Complications (%)	Mortality (%)
Operating room	8 (20.48)	—
UCI	6 (15.36)	1 (2.56)
Out of UCI	**5** (12.8)	1 (2.56)

	*N* = 19 (48.6%)	*N* = 2 (5%)

**Table 4:** Complications in UCI versus out of UCI.

	*N* (%)		*N* (%)
IN UCI		OUT UCI	
Acute renal failure	3 (7.68)	Fistula	3 (7.68)
Pneumonia	1 (2.56)	Anastomotic Leak	1 (2.56)
Bleeding postoperative	1 (2.56)		

Mortality	**1** (2.56)	Mortality	**1** (2.56)

	*N* = 6 (15.36%)		*N* = 5 (12.8%)

**Table 5:** Morbidity and Mortality CS + HIPEC.

Author	Téchnique	Primary Tumor	No patients	Morbidity	Mortality
Sugarbaker [23] 1996	Open + Posop	Appendix Colon	60	35% Anastomotic leak Intestinal perforatión bleeding, biliar leak	5%
Loggie et al. [32] 2000	Close	Appendix colon Stomach	84	30% intestinal leak sepsis, prolonged intubation	6%
Park et al. [33] 1999	Close	Peritoneal Mesothelioma	18	30% infection, pancreatitis	0%
Cavaliere et al. [34] 2000	Open	Ovarian, colon peritoneum, Appendix	40	40% Anastomotic leak, abscess and bleeding	12.5%
Sarnaik et al. [35] 2003	Open	Appendix, Colon Sarcoma, Stomach	33	27 abscess pulmonary embolism, DVT	0%
Fujimura et al. [36] 1999	Expanded peritoneal cavidity	Colon, ovarian, cervical, smallintestine	25	8% Bleeding, abscess	0%
López-Basave et al. [31] 2011	Close/Open	Colon, ovarian, Appendix, pseudo mixoma, Stomach	24	37%Bleeding, fistula pneumonía, renal failure, diaphragmatic opening	0%

**Table 6:** Morbidity and mortality after cytoreductive surgery and hyperthermic intraperitoneal chemotherapy.

Año	Pats	PSM	Death	Morbi	Morbi dity	Common complications		
	(*n*)		(%)		Major	1st	2th	3th
Shen et al. [29], 2004	77		12	—	30	Haematological		
Glehen et al. [27], 2003	216	Mixed	3.2	30.5	—	Intestinal perforation Anastomotic leak	Haematological	Íleus
Kusamura et al. [3], 2006	209	Mixed	0.9	—	30.5	Intestinal perforation/Anastomotic leak	Bleeding	Septicemia
Smeenk et al. [20], 2006	103	PMP	11	54	—	Infection	Intestinal perforation/Anastomotic leak	Cardiopulmonary
Gusani et al. [2], 2008	124	Mixed	1.6	56.5	54	Reoperation	Intestinal perforation/Anastomotic leak	Septicemia
Sugarbaker et al. [19], 2006	356	PMP	2.0	74.2	56.5	Hematological	Gastrointestinal	Cardiovascculary
Chen et al [26], 1997	42	Mixed		86	24	Atelectasis	Pleural effusion	Pulmonary edema pneumotorax pneumonia
Elias et al. [15], 1999	106	Mixed	4	—	41	Intestinal perforation/Anastomotic leak	pulmonar infection	—
Levine et al. [37], 2007	501	Mixed	4.3	43.1	—	—	—	—
Verwaal et al. [14], 2004	102	Mixed	7.8	65	43.1	Intestinal perforation/Anastomotic leak	Infection	Hematological
López-Basave et al. [31], 2011	24	Mixed	0	37		Diaphragmatic opening	Bleeding	Fístula, pneumonia, Renal failure
